# Supplementary material for: Strengthening COVID-19 pandemic response coordination through public health emergency operations centres (PHEOC) in Africa: Review of a multi-faceted knowledge management and sharing approach, 2020–2021
Source: PLOS Glob Public Health. 2023 Jun 22;3(6):e0001386. doi: 10.1371/journal.pgph.0001386 (PMC10286958; doi:10.1371/journal.pgph.0001386)
Supplement: S1 Table — (PDF) [file pgph.0001386.s001.pdf]

S1 Table: Webinar thematic areas, topics by attendees, 2020-2021

Thematic and sub-topic	*# Registrants*	*# Attendees*	*%*
Incident leadership	**15,785**	**2,940**	**23.12**
Decision-making	1,806	268	2.11
Effective communication	1,825	262	2.06
Ethics in emergencies	697	177	1.39
Incident Action Plan (IAP)	104	59	0.46
Incident Leadership	498	220	1.73
Inspiring teams	1,831	193	1.52
Management by Objectives	1,818	218	1.71
Multi-sectoral coordination	196	132	1.04
Rapid Response Team mgt.	1,057	305	2.40
Situational awareness	1,815	258	2.03
Staff health and wellbeing	1,420	302	2.38
Task Management	218	91	0.72
Team building and mgt.	1,830	221	1.74
The PHEOC and Info mgt.	670	234	1.84
Incident Management System	**25,189**	**2,841**	**22.34**
Admin Function	2,742	287	2.26
Country IMS experience	3,572	323	2.54
Implementing IMS	1,673	211	1.66
IMS: Roundup	4,084	466	3.66
Introduction to IMS	1,624	227	1.79
Logistics Function	2,756	266	2.09
Operation Function	2,408	324	2.55
Plans Function	2,570	268	2.11
The Management Function part 1	1,690	189	1.49
The Management Function part 2	2,070	280	2.20
Information management	**9,055**	**1,199**	**9.43**
Data governance	1,861	201	1.58

Thematic and sub-topic	# Registrants	# Attendees	%
Emergency mgt. and critical info	2,033	232	1.82
Introduction to info mgt.	1,847	190	1.49
PHEOC info products	399	118	0.93
PHEOC tools part 2	796	188	1.48
Watch mode operations: EBS	2,048	223	1.75
Watch mode operations: EBS tool	71	47	0.37
Information systems for operationalizing the PHEOC	**3,409**	**379**	**2.98**
PHEOC Info systems part 1	1,701	268	2.11
PHEOC Info systems part 2	529	74	0.58
PHEOC Info systems part 3	585	25	0.20
PHEOC Info systems part 4	594	12	0.09
Learning lessons from PHEs	**4,802**	**621**	**4.88**
Capturing lessons: COVID-19 IAR	1,600	217	1.71
Reflecting on the past to influence the future: Learning from emergencies	1,592	193	1.52
Translating lessons learnt into Actions	1,610	211	1.66
Logistics and Resource mgt. in emergencies	**15,426**	**1,491**	**11.73**
Logistics: Resource mgt.	4,125	310	2.44
Logistics: Round-up session	4,134	332	2.61
Logistics: The Logistician	4,092	322	2.53
Logs. and supply chain mgt. part 1	1,531	288	2.27
Logs. and supply chain mgt. part2	1,544	239	1.88
Multi-sectoral Coordination	**7,172**	**1,136**	**8.93**
Developing and using CONOPs in PHEOCs	1,565	217	1.71
DRR and Health emergency risk mgt.	1,080	217	1.71
Multi-sectoral coordination framework	1,429	243	1.91
One-Health and Multidisciplinary approaches to info mgt.	1,547	226	1.78
Role of law in emergency management	1,551	233	1.83
PHEOC Handbook and Legal Framework	**10,268**	**1,837**	**14.45**
PHEOC Handbook and Legal Framework part 1	4,195	382	3.00

Thematic and sub-topic	# Registrants	# Attendees	%
PHEOC Handbook and Legal Framework part 2	1,312	551	4.33
PHEOC Handbook and Legal Framework part 3	1,534	292	2.30
PHEOC Handbook and Legal Framework part 4	1,594	281	2.21
PHEOC Handbook and Legal Framework part 5	1,633	331	2.60
PHEOC Live tour	**4,124**	**271**	**2.13**
PHEOC Live Tour	4,124	271	2.13
Grand Total	**95,230**	**12,715**	**100.00**
