# Supplementary material for: Strengthening COVID-19 pandemic response coordination through public health emergency operations centres (PHEOC) in Africa: Review of a multi-faceted knowledge management and sharing approach, 2020–2021
Source: PLOS Glob Public Health. 2023 Jun 22;3(6):e0001386. doi: 10.1371/journal.pgph.0001386 (PMC10286958; doi:10.1371/journal.pgph.0001386)
Supplement: S2 Table — (PDF) [file pgph.0001386.s002.pdf]

S2 Table: Frequency of attendance, Webinar series, 2020-2021

Frequency of attendance	Number of participants	Return Rate
28 and above times	179	1.5
20 to 27 times	797	6.6
14 to 19 times	910	7.6
7 to 13 times	2,877	23.9
2 to 7 times	5,367	44.6
Once	1,896	15.8
