# Supplementary material for: Strengthening COVID-19 pandemic response coordination through public health emergency operations centres (PHEOC) in Africa: Review of a multi-faceted knowledge management and sharing approach, 2020–2021
Source: PLOS Glob Public Health. 2023 Jun 22;3(6):e0001386. doi: 10.1371/journal.pgph.0001386 (PMC10286958; doi:10.1371/journal.pgph.0001386)
Supplement: Attachment — Submitted filename: Response to editorial and Reviewers.docx [file pgph.0001386.s004.docx]

**Response to editorial and Reviewers’**

Many thanks for your constructive feedback, here below we illustrate how we have tried to address all the comments to improve our manuscript.

PGPH-D-22-01832

Strengthening response coordination through public health emergency operations centers in Africa: Lessons learned from 56-week webinar sessions, 2020-2021

PLOS Global Public Health

Journal Requirements:

1. Please amend your online Financial Disclosure statement. If you did not receive any funding for this study, please simply state: “The authors received no specific funding for this work.”

The financial disclosure statement was updated in the manuscript accordingly as – The authors received no specific funding for this work.

2. Please update your online Competing Interests statement. If you have no competing interests to declare, please state: “The authors have declared that no competing interests exist.”

Competing interest statement is updated accordingly in the manuscript as – The authors have declared that no competing interests exist.

3. In the online submission form, you indicated that "Data could be accessed upon request to the author". All PLOS journals now require all data underlying the findings described in their manuscript to be freely available to other researchers, either 1. In a public repository, 2. Within the manuscript itself, or 3. Uploaded as supplementary information.

This policy applies to all data except where public deposition would breach compliance with the protocol approved by your research ethics board. If your data cannot be made publicly available for ethical or legal reasons (e.g., public availability would compromise patient privacy), please explain your reasons by return email and your exemption request will be escalated to the editor for approval. Your exemption request will be handled independently and will not hold up the peer review process, but will need to be resolved should your manuscript be accepted for publication. One of the Editorial team will then be in touch if there are any issues.

Relevant data are already included /available in the manuscript itself and optionally uploaded as supplementary information and could be available in a public repository to other researchers.

4. We do not publish any copyright or trademark symbols that usually accompany proprietary names, eg (R), (C), or TM (e.g. next to drug or reagent names). Please remove all instances of trademark/copyright symbols throughout the text, including © on page 7.

Removed the symbol and no copyright/trademark symbols in the document.

5. Please include a separate legend for each figure in your manuscript.

A separate Legend for each figure is included and placed at the end of the manuscript as follows;

**List of Figures**

Figure 1. Webinar Series thematic areas and topics, 2020-2021

Figure 2. Webinar individual topics and Attendees, 2020-2021

Figure 3. Webinar Global and African continent Attendees, 2020-2021

Figure 4. Webinar attendees top represented countries, 2020-2021

Reviewers' comments:

Reviewer's Responses to Questions

**Comments to the Author**

1. Does this manuscript meet PLOS Global Public Health’s [publication criteria](https://eur03.safelinks.protection.outlook.com/?url=https%3A%2F%2Fjournals.plos.org%2Fglobalpublichealth%2Fs%2Fcriteria-for-publication&data=05%7C01%7Cetengw%40africa-union.org%7Cccd546993fb0447f61ce08daeca46367%7C4f56f714477c44a1a135a2fb27680aaf%7C0%7C0%7C638082488042186928%7CUnknown%7CTWFpbGZsb3d8eyJWIjoiMC4wLjAwMDAiLCJQIjoiV2luMzIiLCJBTiI6Ik1haWwiLCJXVCI6Mn0%3D%7C3000%7C%7C%7C&sdata=mJERAkFWZ8SHxkv7XReDNqT4GNjdIm77o53qs0Nd2po%3D&reserved=0)? Is the manuscript technically sound, and do the data support the conclusions? The manuscript must describe methodologically and ethically rigorous research with conclusions that are appropriately drawn based on the data presented.

Reviewer #1: Yes

Reviewer #2: Partly

2. Has the statistical analysis been performed appropriately and rigorously?

Reviewer #1: Yes

Reviewer #2: Yes

3. Have the authors made all data underlying the findings in their manuscript fully available (please refer to the Data Availability Statement at the start of the manuscript PDF file)?

The [PLOS Data policy](https://eur03.safelinks.protection.outlook.com/?url=https%3A%2F%2Fjournals.plos.org%2Fglobalpublichealth%2Fs%2Fdata-availability&data=05%7C01%7Cetengw%40africa-union.org%7Cccd546993fb0447f61ce08daeca46367%7C4f56f714477c44a1a135a2fb27680aaf%7C0%7C0%7C638082488042186928%7CUnknown%7CTWFpbGZsb3d8eyJWIjoiMC4wLjAwMDAiLCJQIjoiV2luMzIiLCJBTiI6Ik1haWwiLCJXVCI6Mn0%3D%7C3000%7C%7C%7C&sdata=pLieTHANyk0BsWWbQ2ypLK2qwO91uRBFrVyEvGBN1VQ%3D&reserved=0) requires authors to make all data underlying the findings described in their manuscript fully available without restriction, with rare exception. The data should be provided as part of the manuscript or its supporting information, or deposited to a public repository. For example, in addition to summary statistics, the data points behind means, medians and variance measures should be available. If there are restrictions on publicly sharing data—e.g. participant privacy or use of data from a third party—those must be specified.

Reviewer #1: Yes

Reviewer #2: Yes

4. Is the manuscript presented in an intelligible fashion and written in standard English?

PLOS Global Public Health does not copyedit accepted manuscripts, so the language in submitted articles must be clear, correct, and unambiguous. Any typographical or grammatical errors should be corrected at revision, so please note any specific errors here.

Reviewer #1: Yes

Reviewer #2: Yes

5. Review Comments to the Author

Please use the space provided to explain your answers to the questions above. You may also include additional comments for the author, including concerns about dual publication, research ethics, or publication ethics. (Please upload your review as an attachment if it exceeds 20,000 characters)

Reviewer #1: This is an interesting paper, informative and well written. However I feel a little more analysis and some slight edits are needed

Line 122: data extraction too (correct to tool)

Corrected the typo error accordingly – tool.

Line 194 - 197: A table // annex describing what these thematic / topics are and contain would be helpful. It isn't clear what is contained in PHEOC doc part 2 for example

The various topics covered over the 56 weeks sessions are shown in Figure 4 against the number of attendees per each topic. However, to address the suggestion and provide more information, annex is added containing the thematic areas, topics covered under each series, number of registrants, and attendees per topic.

Line 212 - Please describe what a 'discord platform' is

A description is provided in the manuscript.

Line 251 - Nigeria and Ethiopia participation - authors should consider whether the populations of the countries (the two biggest in Africa) is a part explanation for the large participation. Further reflection on why such large US participation since US was not a target

High participation in the webinars does not necessary a corresponding number of public health emergency management staff in country. The high participating could be resulted from the presence of Africa headquarters where there is high chance deploying more staff in HQ and partner connections with Nigeria CDC, Ethiopian Public Health Institute. These are likely to have more of an influence on the number of attendees from these countries. Though the primary target audience was Africa, the webinar was extended to address the global audience as it was the only webinar covering PHEOCs. Similarly, the high participation of the US could be due to the level of awareness in the country and advertisements made through US CDC and Resolve to Save Lives (RTSL) as these were part of the working group. This has been somehow explained in the discussion part.

Line 269-279 - the sentences here seem incomplete

We reread the sentences and made the sentences complete.

General

Some information on languages used for delivering the webinars and reflection on the impact on participation would improve this review. Africa Union has 5 official languages, what proportion of sessions were delivered in English, French Arabic, Portuguese etc? A correlation between language of delivery and country participation may be informative

Impact on practice - some comment on the impact of the webinar series on actual practice - operationalization of PHEOCs would be informative. If this has not been done, perhaps the authors could indicate why not and how they propose to do this in the future to inform further development of the programme?

Reviewer #2: Thank you for the opportunity to review this paper, which examines the implementation of a knowledge support service by an inter-agency team during the COVID-19 pandemic. The intervention aimed to strengthen COVID-19 response of Public Health Emergency Operations Centres.

The adaptation of knowledge support and information dissemination practices are critical changes that took place during the pandemic, and which have had wide-reaching implications for public health interventions and crisis response. The intervention that is presented in this paper can provide an important addition to the literature, to improve our understanding of how to effectively adapt in a changing context with movement restrictions. The evaluation presented can help to inform agencies and organisations seeking to do similar work.

At present, the organization of the paper and also the content needs some revision to be able to provide clear evidence. In its current format, the manuscript provides a mix of information in the introduction, methods, results, and discussion, with some results described in the introduction, the methods are very short, some methods are described in the results, and some results are presented for the first time in the discussion. Also, the title implies that the paper reviews the webinar series, but the paper provides some interesting information about a Community of Practice, which is useful and important to share. The title should be revised to reflect this or perhaps to describe a multi-faceted intervention to share knowledge and experiences for teams engaged in the COVID-19 response (e.g. webinar series and CoP).

Another key issue is that the findings presented do not examine quality and it isn’t clear if it was possible to examine effectiveness or impact of the webinars (e.g. accurate knowledge acquisition and change in practice). In order to receive a certificate, participants had to attend all webinars and also complete a knowledge check. If the results of the knowledge check are available, this would help to provide some insight into quality.

The examination of the results and limitations is currently a bit superficial with descriptive statics, and the conclusions about effectiveness aren’t supported by the presented findings. I’ve provided some detailed comments below which I hope will be helpful.

Overall organization – The introduction should be used to provide information about the context and the need/justification for developing the intervention. The methods should describe what the author group has done (e.g. how was the intervention assessed/evaluated?). The results section should describe the intervention itself and any analyses that were possible. All components of the intervention – including the development of the Community of Practice (CoP), with clear descriptions of the aims and objectives for each component, should be included. The discussion should unpack the meaning of the results and consider findings of other relevant studies or interventions. It might also be helpful to provide a timeline which indicates the declaration of the pandemic and key interventions that this study examines (forming of secretariat, intervention planning, webinar start date, set up of CoP, start date of including participants’ inputs into the topics, start date of introducing language translation (if done), webinar finish date). It is also necessary to clarify who the different actors and contributors are. Is the PHEOC working group specific to this webinar series? Or the CoP? Or is it broader?

Page 4 line 122 – typo. “Data extraction tool” is missing an l at the end of “tool”

Corrected the typo error accordingly – tool.

Page 4 Results – please indicate when the intervention was held (date range of webinars, date or month when the CoP was set up, etc)

The first paragraph of the results section is rephrased and put below to address the comment and provide informative information. A total of 56 webinar sessions were held between June 2020 and December 2021, where the first five sessions were conducted on Thursdays, starting at 3:00 p.m. East Africa Time (EAT). However, an adjustment was made later to accommodate more participants and pushed the start time to 4 p.m. EAT. Of the webinar period.

Page 5-6 Table 1 – for the discussion, it would be interesting to unpack the overlaps in the “Summary of Challenges” and “Areas of Good Practice” as several topics appear in both.

Page 6 lines 160-162 – this information is presented earlier. Suggest to remove the earlier information and keep it here in the results section

The statement was rephrased to only indicate the establishment of a secretariat – as a secretariat was established to oversee overall webinar delivery. The statement in lines 160-162 is kept.

Page 6 lines 170-174 – quality assurance. This is a crucial topic that should be significantly expanded. It is particularly important in the rapidly evolving context of the pandemic. Did the organizers succeed in obtaining and reviewing all presentations before they were given? Was the terminology kept consistent? Or were corrections made during the course of individual webinars? Were there any challenges in the quality assurance activities? Any gaps? Lessons learned?

Yes, we made it clear in the manuscript. The webinar coordination group was responsible to ensure quality of the presentation, ensure consistency with existing guidance and policy. Presenters were required to submit their final presentation for review by the group before presenting it.

Page 6 lines 175-183, Webinar delivery – was language translation done? It sounds like it may have been but it isn’t clear for which languages and the text implies it wasn’t done for all the sessions. This is a huge issue for reach and comprehension – and therefore quality, safety and effectiveness of the intervention – and should be addressed. Also were the surveys sent in multiple languages? Does the CoP have some support for translation or what is/are the functional languages?

Initially, the webinars were being deliver in Eglish, however, translation services in English, French and Arabic were provided as the webinars progressed.

Page 7 line 187 – who are the public health actors at country level? Please clarify which stakeholders were include in the design and content of the webinars

The public health actors who were part of the design and content of the webinars are either regional, headquarters, and international organizations as described in the introduction part, page 2, lines 86-91. public health actors at the country level that include country offices of the regional and headquarters actors who were part of the design and content development (e.g., WHO, Africa CDC, and WAHO), local and international stakeholders including national public health institutions (NPHIs), NGOs, etc that are supporting emergency management activities at country level. In fact, we made some changes in the statement to mention some of the key actors who were part of the process.

Page 7 line 189 – were the deep dive format of webinar for 1.5-2 hours enough to provide a “thorough” understanding? This statement will need to be defended or the language should be altered.

The statement aims to explain this – to enable participants to have a thorough understanding of each topic, webinar topics were reorganized/restructured into webinar series where various topics are identified and covered in subsequent sessions under respective series. For example (Fig 3), the webinar series multi-sectoral coordination was delivered in 5 sessions, each session focused on a certain topic that falls under the series. We believe that reorganizing the topics by thematic area and focusing on a specific area in each session for 1.5-2 hrs is adequate time to allow participants to have a good understanding. However, some edit has been made to the statement to increase clarity – The prioritized topics were reorganized into a series where various topics were covered in subsequent sessions under each series to allow participants to take a deep dive into the content and gain a thorough understanding of each topic.

Page 7 paragraph 3 – the contents of this paragraph would be more effectively presented in a table. The table should list all the themes (column 1) and all the subtopics for each theme (column 2). It would help to also provide an additional column with number and % participants for each subtopic to get an idea of uptake and interest. Also consider including a column for the number of people who registered, to look for patterns in registration vs attendance. The text should examine the table and the patterns in uptake to help characterize the update and participation.

We could still agree to include a table or annex having a column covering the variables as suggested and initially thought to summarize the key information in the table, but it was bulky and replaced with a figure (Fig 4) showing the list of topics over the 56-week sessions and the number of attendees. However, to address the suggestion and provide more information, a description of topics covered under each thematic series is provided in Annex 1 containing the thematic areas, topics covered under each series, number of registrants, and attendees per topic.

Page 7 – post-webinar survey- why was the period of post-webinar surveys limited? Why wasn’t it done for all of them? Was it delivered in multiple languages?

The post-webinar survey was started during webinar 16 containing six questions and later one question to account for their role in PHEOC was added starting webinar 30. There is no as a such major reason why the post-webinar surveys started later however during the first few webinars, the core working group was a bit overwhelmed setting the scene, planning and organizing the delivery of webinars, etc. later, it was agreed to initiate a survey aiming to assess topic relevance, time for discussion, etc.

Page 8 paragraph 1 (Interaction and networking within the online CoP) – this paragraph is difficult to follow and should be reworked to clarify and improve the flow of the text. As mentioned above – this is a separate component of the intervention – with a different aim, different objectives and different delivery/management than online webinars and perhaps also a different time period (is the CoP still active?). Also, please explain with the “Discord Platform” is, as this is not widely known.

We have reviewed, reworked, and improved the descriptions/summary under this section.

Page 8 Reward on Participation – the knowledge check is briefly mentioned here. If available, the findings/scores of the knowledge check should be presented to provide some idea of participant knowledge. If any pre-tests were done, then the pre- and post-tests should be compared.

The knowledge check/quiz was given to those who completed at least four webinar sessions to allow them to get a certificate if they score a pass rate of 80%.

Page 8 Discussion lines 231-2 – this is the first place where the reader learns that the webinars were biweekly. This should be put into the results in a description of the intervention.

We agreed and added a statement in the results section to show the frequency of the webinars (weekly and bi-weekly).

Page 8 line 236 “outreached” – this is an unusual use of this term, would be more effective to rephrase. Suggest “reached beyond the intended primary audience” or something to that effect.

It is reworded from outreached to reached beyond as suggested and the statement modified accordingly as – reached beyond the intended primary audience.

Page 8 line 237 – this conclusion is not supported by the data. Participants’ perceptions suggest the webinar strengthened knowledge but there isn’t any objective evidence to prove this happened. There is also no clear examination of the CoP impact on knowledge, access to information or networking.

We now reviewed and updated the conclusion part based on the new changes we made to the document and aligned with the findings.

Page 9 paragraphs 2-5. The discussion should critically examine the patterns in the findings, such as the changing pattern in attendance. There appeared to be higher uptake, then a lull and then a steady increase later on (Figure 1). Why did this happen? What changes were made in implementation that might have affected this? How could communication or advertising have impacted it? What about language, any concurrent discussions in the CoP, or the ability of participants to influence the topic? These and other factors may have affected participation in different seminars; it would be helpful to understand whether or not that might be the case and any evidence to support.

The discussion is reviewed to address the key findings and their meaning.

Page 9 lines 264-265 – Is there evidence that inviting countries to share experiences in this intervention helped other countries learn, adapt and engage? If so, it should be presented here. All conclusions must be supported by the findings.

There was no really impact study done to show how other countries benefited by the experiences shared by other countries. The conclusion no is reviewed to ensure they are drawn from the findings.

Page 10 paragraph 2 – the second half of the paragraph presents the findings and should be put into the results section

We now reviewed all the paragraphs in all sections to ensure the organization and flow.

Page 10 paragraph 3 – were these surveys done in English? How would that affect the reach and validity of the findings?

The survey was actually in English, however since the questions were brief and we believe the professionals could understand them. However, to ensure validity of interpretation of the findings, this something we need to consider for future.

Page 10 lines 290-291 – the sentence is unclear, please rework to clarify

The statement is rephrased to clarify that there are similar findings from other surveys where participants agreed link for registration to webinars were shared in advance and the topics were relevant. To reflect this, the statement is modified as - Similar reviews found that 89% of respondents agreed that webinar organizers provide links for registration well in advance, and 91% agreed that webinars focused on relevant topics.

Page 10 lines 293-295 – This implies success when there appears to be variable uptake of the webinars. This is a good space to critically examine the findings.

We reviewed the sentences accordingly to the new changes we made to the manuscript.

Page 11 – Limitations – this section should be markedly expanded. My previous comments allude to a number of limitations that should be described. An especially important limitation is the lack of measurements for quality and impact of the webinars as well as the CoP. It’s a huge challenge for all knowledge and capacity strengthening programmes, whether online or in-person, and should be unpacked here and any activities/adaptations that were made to address the limitations should be described. Please also note that the survey is likely to be prone to response bias and pleasing.

We agree there are a few limitations in measuring the quality and impact of the webinars. As described in the introduction and results section, however, efforts were made to address quality issues including the establishment of a working group to oversee the delivery of webinars including assessing the quality of the presentations and ensuring they are prepared according to the template addressing key areas. Components. Besides, presenters were selected from different organizations based on their expertise in the subject area. In the future, quality assurances and the impact of webinars should be considered, and specific variables need to be addressed in survey questions. We modified the limitations section to address the input.

Page 11 – Conclusions, line 315-318 – the findings presented do not support this conclusion. If there are data to support the conclusion, this should be included in the results and unpacked in the discussion.

we now went through the conclusion, reworked and drawn the conclusion based on our findings.
