# Supplementary material for: Strengthening COVID-19 pandemic response coordination through public health emergency operations centres (PHEOC) in Africa: Review of a multi-faceted knowledge management and sharing approach, 2020–2021
Source: PLOS Glob Public Health. 2023 Jun 22;3(6):e0001386. doi: 10.1371/journal.pgph.0001386 (PMC10286958; doi:10.1371/journal.pgph.0001386)
Supplement: Attachment — Submitted filename: Rebuttal letter response to Reviewers.docx [file pgph.0001386.s005.docx]

**Rebuttal letter – Responses to Reviewers and Editorial**

Many thanks to the reviewers and editorial team for very insightful comments and feedback. We have attempted to address all the comments to improve our manuscript, as shown below.

Journal Requirements:

1. Please review your reference list to ensure that it is complete and correct. If you have cited papers that have been retracted, please include the rationale for doing so in the manuscript text, or remove these references and replace them with relevant current references. Any changes to the reference list should be mentioned in the rebuttal letter that accompanies your revised manuscript. If you need to cite a retracted article, indicate the article’s retracted status in the References list and also include a citation and full reference for the retraction notice. We made changes to the reference, for example, included link to access the references.

2. In the online submission form, you indicated that "Data could be accessed upon request to the author". All PLOS journals now require all data underlying the findings described in their manuscript to be freely available to other researchers, either 1. In a public repository, 2. Within the manuscript itself, or 3. Uploaded as supplementary information. This policy applies to all data except where public deposition would breach compliance with the protocol approved by your research ethics board. If your data cannot be made publicly available for ethical or legal reasons (e.g., public availability would compromise patient privacy), please explain your reasons by return email and your exemption request will be escalated to the editor for approval. Your exemption request will be handled independently and will not hold up the peer review process, but will need to be resolved should your manuscript be accepted for publication. One of the Editorial team will then be in touch if there are any issues. Major figures are included in the manuscript itself and additionally, we have now uploaded the data as supplementary information.

**Reviewer #3:** It is important to have this abbreviation PHEOC in the title as it appears in the short title => Strengthening COVID-19 pandemic response coordination through public health emergency operations centers (PHEOC) in Africa: Review of a multi faceted knowledge management and sharing approach, 2020-2021 Agreed and added the abbreviation as suggested.

Use the same language (British English or American English). For instance, the word “Centers” in the title but written as “Centre” in the key words section. We are now using British English throughout the document for consistency purposes.

Abstract:
There must be an omission in the following sentence: In-person events including training workshops and on-site technical support were to limit the spread of the ??? then fast-spreading COVID-19 pandemic. Or good to delete the word “then”. Corrected with adding a (-) between then and fast. The idea here is we are referring to the rapid spread/evolution of the COVID-19 pandemic during the initial period.

Method: Put full stop after the first sentence. Corrected (full stop added).

The abbreviation PHEM is not defined at its first appearance. Corrected (full stop added).

Introduction:
There is a need to define what is a functional PHEOC and what are the criteria set to judge it functional. A description is added in the second paragraph of the introduction to define what key components need to be in place to have a functional PHEOC.
The text from line 86 to 112 seems describing the methods used and could be in the Method section. The information provided here is to provide the context of the webinars.

At line 122, the word “too” could read as “tool”. This has been already corrected in the revised manuscript.

There is need for a specific paragraph describing the objective and research question for this study. We have made the objective clear in the last paragraph of the introduction and added some sentences in the fourth paragraph of the introduction on the research question to elaborate.
Methods
Well described for the process used. However, the study design is not clear. Is it a qualitative, quantitative, or mixed? It does not give details on how the data were analysed. Any test used? Statistical software? Database created with what software (STATA, SPSS, … Excel,…)? Was there a needed sample size for this study? How was this calculated? The database was created using MS Excel and data was analysed using Excel and R from which tables, charts and figures were produced.
By reading the following paragraph, we can conclude that it was a mixed method: A thematic analysis (i.e., inductive, and semantic approaches) was used to group, analyze, and describe major findings. Further descriptive statistics (percentages, tables, charts, etc.) were used to summarize the quantitative data. However, when reading the table 1, it shows figures. The design should be clearly stated and described in detail. The thematic approach used should be clear as the manuscript is already written, it should not say i.e. We have worked on this section in the manuscript to clarify the process.

The Ethics statement is not detailed. As this includes live participants who responded to questions that were included in the analysis, there is a need to know if these participants gave their consent for their answers to be involved in any publication. Were they aware that these questions were asked for the purpose of research? We have added more information in this section (page 5). No personal information was collected, and survey respondents were informed about the study’s aim and knew that by responding they’d consent to participation.

Results
The lines 153 to 155 should be in the “Methods” section. The lines 170 to 189 should be in the “Methods” section. The following sentence: In addition to this Africa, CDC had IT support… should read as: In addition to this, Africa CDC had IT support… At the line 204, it is good to clarify what the additional question is. In the table 1 (line 206), why percentages of No answers are in italic while they are not for Yes?

Line 210: The following sentence: In the individual webinar sessions, there were, on average, 26 interactions between the participants and facilitators, ranging from 4 to 56 interactions per session. should read as: On average, there were 26 interactions between the participants and facilitators in the individual webinar sessions, ranging from 4 to 56 interactions per session. This has been already corrected in the revised version.

It could be better to list some examples of best practices that were exchanged between countries through the webinars and understand how these were implemented where they are lacking to improve the response to the pandemic. Please include some practices that each PHEOC learned from each other.

Line 273: The sentence: Organizers recognized that participants tended to drop off the webinar after one hour, likely because of conflicting commitments, therefore, it is recommended that future webinar sessions do not extend beyond one hour. should be split into 2 and should read as Organizers recognized that participants tended to drop off the webinar after one hour, likely because of conflicting commitments. Therefore, it is recommended that future webinar sessions do not extend beyond one hour. Corrected as suggested (the sentence is splitted into two).

Line 293: This sentence is too long: The success of the topic choice and strong communication of webinar sessions in advance is indicative of the commitment of the PHEOC Network partners, PHEOC Webinar working group, and secretariat, and emphasizes the effort involved in conducting a weekly, interactive webinar series, especially when hosted by SME’s whom themselves were engaged in COVID-19 response activities. and can split into 2 as: The success of the topic choice and strong communication of webinar sessions in advance are indicative of the commitment of the PHEOC Network partners, PHEOC Webinar working group, and secretariat. They emphasize the effort involved in conducting a weekly, interactive webinar series, especially when hosted by SME’s whom themselves were engaged in COVID-19 response activities. Corrected as suggested (we have splitted the sentence).

Line 304: This sentence is too long: The interactions observed appear to be low as compared to the number of attendees per session (235 on average), which could be due to the limited time allocated for discussion, however other considerations may include the likelihood of attendees listening to the session while conducting other work, language barriers or their ability or confidence to engage with the other attendees and facilitators in a virtual environment. and can split into 2 as: The interactions observed appear to be low as compared to the number of attendees per session (235 on average), which could be due to the limited time allocated for discussion. However, other considerations may include the likelihood of attendees listening to the session while conducting other work, language barriers or their ability, or confidence to engage with the other attendees and facilitators in a virtual environment. Corrected as suggested (the sentence is splitted into two).

Conclusion
Line 321: This sentence is too long: Besides, there was an overall low attendance rate though the high number of registrants indicates interest and acceptance of the Webinar Series, and almost half of the participants came from just a few countries, possibly explained by biased in the advertisement of the webinars based on partners involved. and can split into 2 as: Besides, there was an overall low attendance rate though the high number of registrants indicate interest and acceptance of the Webinar Series. Almost half of the participants came from just a few countries, possibly explained by biased in the advertisement of the webinars based on partners involved. We have adjusted this in the revised manuscript.

It could good to see how this program can be made sustainable and improved to serve as model for future health emergencies.

**Reviewer #4:** I am using the "Revised Version" of the manuscript for this review and am editing the text based on the accompanying Line Numbers.

GENERAL COMMENTS:

This article describes an innovative set of interventions to improve staff knowledge and overall public health capacity in Africa, as a response to the novel COVID-19 pandemic. It provides relevant information on the processes, and the evolving approaches over time, in response to participant feedback.

A major statistical issue that needs to be addressed is the percentage level of participation. The authors need to be more clear and precise as to what is the denominator that is being used to estimate the different types of participation. In the paragraph titled Webinar participation (Lines 228 to 242) you state that a total of 95,230 participants registered the the Webinar interventions. I assume that this is the denominator that measures levels of participation for many of the citations. However, this explanation doesn't appear until late in the paper. In addition, can you better define this? It would be helpful to describe how you advertised this approach broadly and how various public health persons signed onto an active e-mail list that was then used to notify participants of upcoming sessions and the availability of materials. I would recommend that you provide a short summary in the ABSTRACT of how this overarching denominator was established and then at the beginning of the METHODS section provide more detail. The relevant denominator becomes confusing, because in the Abstract and early in the Main article you cite that 112,354 persons interacted with Zoom and other webinar resources. Were these discrete individuals that had enrolled onto an e-mail contact list? Many readers will confuse this number with the registered participants number. I would recommend that you remove the 112,354 citation in the abstract and instead present the number of persons who registered for the approach (95,230)

Another major issue is that the article relies on participant self assessment to address the overall impact of the approach. It would be helpful in the Discussion session to briefly consider and present possible methods in future evaluations of this approach for a more impartial assessment as to whether public health services were actually improved. Did the quantity and quality of services get better and in what ways? Was there any impact on incidence, standards of care, patient outcomes, etc, etc??

SPECIFIC COMMENTS:

I have made multiple recommendations below, using the line numbering, to address spelling, grammatical, and technical issues.

ABSTRACT

In Line 40, replace the word, "through" with "utilizing." Corrected.

In Line 54-55, the final phrase is awkward and needs to be rewritten. For example, "...and the simplicity of the delivery of the training, through interactive webinairs and a community of practice, encouraged a greater number of public health staff to participate, and to spread the word of the training to their own networks." Rephrased the sentence as suggested to improve clarity of the message.

In Line 45 remove the sentence, " Major findings were synthesized and described per thematic area". You have duplicated the previous sentence. Corrected – removed the sentence.

In Lines 48 to 50 the authors refer to "112,354 persons interacted with the sessions across ZOOM, Facebook and You tube......" This is confusing because the reader may assume that this is a denominator for participation. But it isn't. The denominator is actually 95,230 participants that were registered to be part of the approach. Per my note at the beginning of this Comments section, it would be helpful if you provide information on the denominator for participation earlier in the document and remove the 112, 354 number from the Abstract section. We have addressed this in the revised version.

INTRODUCTION:

In lines 66-67, revise to "...abruptly interrupted, testing the recent increases in health security investments that African countries had received since the end of the West African Ebola outbreak in 2016." Rephrased the sentence as suggested.

Line 92, add "events" after face-to-face" Corrected as suggested.

Line 93, replace "global" with "globally" Corrected as suggested.

Line 94 replace "till" with "still." Corrected as suggested.

In line 97, replace "suing" with "using." Corrected as suggested.

In line 99, replace "...consiting of creating and online..." with "and included the creation of a Community of Practice (CoP, comprising mainly PHEOC professionals fro the African continent." Corrected as suggested.

In Line 104, can you footnote the term "Discord" and provide a short description of the application? A footnote was added to elaborate on what a discord means … *An open-source social communications and online community platform capable of hosting a large virtual community, group, and one-on-one messaging, live in-app audio and video meetings and ease of sharing various file formats*.

METHODS

In Lines 120 to 123, can you add any information on the approximated number of staff in the secretariat and any information on the annual budgeting costs?

WEBINAR FACILITATION.

Line 125, rewrite, "The webinar series was initiated to respond to identified gaps in multiple....." Corrected as suggested.

Line 127, can you find a different term for "take -off curriculum?" I don't know what this means. ....." We have rephrased/replace with different words.

Line 137, replace "tour" with "tours" and make "human resource" plural-resources" Corrected as suggested.

Line 145, replace "series" with "multi-part series" Corrected as suggested.

Liness 154-155. Use this sentence which describes DISCORD, as a footnote as noted above for Line 104.

Line 198, replace the word topic with the plural, "topics" Corrected as suggested.

Lines 222-226, as stated in my general comments, the authors do not define the baseline numbers, so the participation percentages are not clear. Please provide the definition of and the actual number of persons who make up the audience for this exercise. Is there an enrollee email address list that was created??? How was this done? We have now defined the denominator.

Line 244, add the word, "with" just before the words, "...webinar 16,..." Corrected as suggested.

DISCUSSION

Overall, I found the DISCUSSION section to be excellent. However, there are some key sections that could be improved.

Lines 307 to 310. This sentence is very confusing. Could you replace it with the following, "However, , the inclusion of key partners of the Africa CDC and US CDC in the PHEOC Webinar Series working group is likely to have resulted in an inordinately large number of of participants from outside the Africa PHEOC network." Corrected as suggested.

Line 320, what is an "MS PHEOC?" Words adjusted as, …. Member States on their coordination through PHEOC….

LIMITATIONS

Lines 367 to 370. I found this section to be extremely confusing. The authors are trying to describe possible biases in the topic selection, but these arguments are not well presented. Can you rewrite this section?? We have worked on and rephrased the limitation section as suggested.

Lined 384. In this sentence the authors refer to the PHEM training. What does the abbreviation PHEM mean? We now provided the definition of PHEM in the manuscript – public health emergency management (PHEM).
**Reviewer #5:**

**Authors:**

- I note the A-CDC director at the time has not been included as a co-author on this paper. While not strictly necessary, it might be worth considering and acknowledging the role of senior leadership in facilitating major pandemic emergency response projects like this – If he agrees to review the paper, that would meet the ICJME criteria for authorship. Womi have added as author after his consensus for review and inclusion as a co-author.

**Abstract:**

- Suggest you revise the first sentence as ‘interrupted *implementation of* program plans’ Corrected as suggested.
- Sentence on A-CDC – suggest revising to reflect this was designed to support *other countries* – the current wording implies it was internal to A-CDC only Corrected as suggested.
- Results – how many countries? Ie ‘1968 members across XX countries’.

**Abstract:**

- The version I received for review had one abstract at the beginning of the pdf and another after the title and authors – they’re not the same, and I’m not sure which one is revised vs original. Please ensure comments above are reflected in the final abstract. We have address comments on our revised abstract and document.
- In this (2^nd^) abstract – please ensure background section is clear that you’re referring to ‘in-person events *across the African continent’* Corrected as suggested.
- Results – any *new* PHEOC’s established as a result of this capacity-building effort?
- Conclusion – the word ‘consequential’ is confusing in this context. Suggest replacing with something more specific

**Introduction:**

- For those not familiar with PHEOCs, you need to add something in the 2^nd^ sentence that clearly outlines that this is a *national level structure* which is ideally embedded in the Ministry of Health (or National Public Health Institute) Corrected as suggested, added a sentence in the second paragraph of the introduction section.

**Results:**

- 2^nd^ paragraph – the pandemic interrupted *implementation* of annual plans Corrected as suggested.
- Table 1 – format as left-aligned, not justified Corrected as suggested.
- Line 208, 212 – use of ‘CoP’ – this was never abbreviated in the previous sections – the full wording was used in a heading but I didn’t see the acronym defined. We have put in full wording in the introduction and methods sections.

**Discussion:**

- ‘bi/weekly’ should be ‘biweekly’ … or ‘(bi)weekly’ if both weekly and biweekly were used Corrected as suggested.
